# Electrochemical Mechanism of Tellurium Reduction in Alkaline Medium

**DOI:** 10.3389/fchem.2020.00084

**Published:** 2020-03-03

**Authors:** Tingjun Wu, Jiwon Kim, Nosang V. Myung

**Affiliations:** ^1^Department of Chemical and Environmental Engineering, University of California, Riverside, Riverside, CA, United States; ^2^Laboratory of Advanced Functional Materials, Xiamen Institute of Rare-earth Materials, Haixi Institute, Chinese Academy of Sciences, Xiamen, China; ^3^Materials Science and Chemical Engineering Center, Institute for Advanced Engineering, Yongin-si, South Korea

**Keywords:** electrochemical kinetics, tellurium reduction, alkaline bath, LSV, EQCM, Tafel slopes

## Abstract

A systematic electrochemical study was conducted to investigate the reduction of tellurium (Te) in alkaline solutions. The effect of various parameters, including tellurite ion concentration, applied potential, and pH was investigated by both linear sweep voltammograms (LSVs) and electrochemical quartz crystal microbalance (EQCM). EQCM was essential to understand the reduction of Te(0) to soluble ${\text{Te}}_{2}^{2-}$(-I) or Te^2−^(-II). The Tafel slopes for two Te reduction reactions [i.e., Te(IV) to Te(0) and Te(0) to Te(-I)] indicated that the electrochemical reduction of Te is strongly dependent on solution pH, whereas it is independent of the concentration of ${\text{TeO}}_{3}^{2-}$. At relatively weaker alkaline solutions (i.e., pH ≤ 12.5), the discharge of ${\text{Te}(\text{OH})}_{3}^{+}$ was determined to be the rate-limiting step during the reduction of Te(IV) to Te(0). For the reduction of Te(0) to Te(-I), the reaction follows a four-step reaction, which consisted of two discharge and two electrochemical reactions. The second discharge reaction was the rate-limiting step when pH ≤12.5 with the Tafel slope of 120 mV/decade. At a higher pH of 14.7, the Tafel slope was shifted to be 40 mV/decade, which indicated that the rate-limiting step was altered to the second electrochemical reaction. Te(0) deposits were found either on the surface of an electrode or in the solution depending on pH due to the different rate-limiting reactions, revealing that pH was a key parameter to dictate the morphology of the Te(0) deposits in alkaline media.

## Introduction

Tellurium (Te), due to its unique crystal structure, exhibits many unique properties, such as piezoelectric effect, photoconductivity, catalytic activity, gas sensing, and thermoelectrics (Bradstreet, 1949; Shurygin et al., 1975; Kambe et al., 1981; Fujiwara and Shin-Ike, 1992; Wang et al., 2006; Liu et al., 2010b; Zhang et al., 2012; Lee et al., 2013; Park et al., 2013). Recently, considerable attention has been also directed to tellurides, such as bismuth telluride (Bi_2_Te_3_), lead telluride (PbTe), antimony telluride (Sb_2_Te_3_), and cadmium telluride (CdTe), revealing remarkable performances in electronic applications (Wuttig and Yamada, 2007; LaLonde et al., 2011; Kumar and Rao, 2014; Guo et al., 2015).

Electrodeposition, which requires ambient conditions and low capital cost, has been widely studied for a synthesis of tellurium and its alloys, and it has been especially promising for large-scale deposition with higher deposition rate to fabricate the Te and its alloy for a variety of applications (Wu et al., 2014, 2015; Ferrer-Argemi et al., 2019; Kim et al., 2019). For instance, the combination of electrochemical deposition of compound semiconductors (metal tellurides) with standard integrated circuit technique makes fabrication of thermoelectric microdevices possible, which can convert rejected or waste heat into usable electric power. The understanding of electrochemical reactions of Te ion in alkaline conditions would benefit the electrodeposition metal telluride compound for thermoelectric microdevices.

In order to apply Te or its alloys for a specific application, the morphology, crystal structure, and size must be precisely controlled to exhibit different properties. Thus, it is critical to understand the relationship between the operating conditions of electrodeposition—such as electrolyte concentration, pH, temperature, and agitation rate—and resulting physical properties of the synthesized Te. In addition, because the satisfactory quality of Te electrodeposits is highly hinged on a critical control of a technique with a detailed understanding of deposition mechanism, it has been emphasized to investigate the reduction mechanisms in a wide range of electrodeposition conditions. The electrodeposition of Te has been investigated extensively in both aqueous and non-aqueous solution to incorporate chemical and electrochemical thermodynamics as well as to understand the kinetics of electrochemical reactions of Te. Most of the works demonstrated the Te electrodeposited with various nanostructures, showing the dimensional effect on the physical properties. For example, the piezoelectric property of one-dimensional (1-D) nanowires was enhanced by reducing the diameter of the nanowire due to the flexoelectric effect (Liu et al., 2010a; Wang, 2012). In addition, the light absorption observed in 3-D Te nanostructures was enhanced due to the refractive index gradient from nanostructures (Diedenhofen et al., 2009; Chao et al., 2011; Yan et al., 2014). These works, however, typically employed acidic electrolytes, which have low TeO_2_ solubility, inevitably leading to low tellurium ion (${\text{HTeO}}_{2}^{+}$) concentration. This would allow for the deposition reaction to be under the ${\text{HTeO}}_{2}^{+}$ diffusion control, and consequently, it would be difficult to macro-structure due to a low deposition rate.

The higher solubility of TeO_2_ in alkaline solutions allows the fabrication of thick compact films (Wu et al., 2014, 2015). The high ${\text{TeO}}_{3}^{2-}$ concentration in the electrolyte would rapidly replenish the ${\text{TeO}}_{3}^{2-}$ near the substrate, improving the deposition rate and uniformity of the electrodeposits. The electrochemical reduction mechanisms of Te in alkaline solutions have been examined by several groups using cyclic voltammetry (CV). Lingane and Niedrach. reported that the ${\text{TeO}}_{3}^{2-}$(IV) was reduced to Te(0), observed by the black deposit on the working electrode, then Te(0) was further reduced to ${\text{Te}}_{2}^{2-}$(-I), correlated to the deep violet color of the solution, which finally changed to colorless, representative of the reduction of ${\text{Te}}_{2}^{2-}$(-I) to Te^2−^(-II) (Lingane and Niedrach, 1949). Schmidt cited the reaction mechanisms from Lingane's publication, but did not mention the formation of ${\text{Te}}_{2}^{2-}$(-I) (Schmidt, 1962). Shinagawa et al. reported the same reduction reaction mechanisms as Lingane and Niedrach. (Shinagawa et al., 1972). Mishra detected the presence of intermediate species ${\text{Te}}_{2}^{2-}$(-I) by using a rotating ring-disk electrode as Te^2−^(-II) was oxidized to Te(0) (Mishra et al., 1990). The Tafel slopes of the reduction reaction [i.e., ${\text{TeO}}_{3}^{2-}$(IV) to Te(0) and Te(0) to Te(-I)] were reported by several groups and possible reaction mechanisms were proposed (Awad, 1961, 1962; Komandenko and Rotinyan, 1966, 1967a,b). Our previous publication also discussed the reaction mechanisms for Te in comparison with the literature data (Wu et al., 2014, 2015, 2017). However, a systematic study of Te reactions in a large Te ion centration and pH window using linear sweep voltammograms (LSVs), electrochemical quartz crystal microbalance (EQCM), and Tafel plots has not been reported. While the Tafel plots provide details about reaction kinetics, EQCM provides detailed information to interpret the mechanism for reduction of ${\text{TeO}}_{3}^{2-}$(IV) and Te(0). Among the valence states of Te, ${\text{TeO}}_{3}^{2-}$(IV), ${\text{Te}}_{2}^{2-}$(-I), and Te^2−^(-II) are solvable species, while Te(0) is solid. Therefore, when the Te(0) is deposited, the mass on the electrode would increase. When the Te(0) is then reduced to either ${\text{Te}}_{2}^{2-}$(-I) or Te^2−^(-II), the solid Te would dissolve and the mass on the electrode would decrease. This mass change can be recorded by the EQCM to understand the electrochemical reactions of Te.

In this work, voltammetric investigations of reduction mechanisms from ${\text{TeO}}_{3}^{2-}$(IV) to Te(0) and from Te(0) to Te^2−^ (-I) were systematically conducted in various alkaline solutions. The effect of tellurite concentration and pH on reduction reaction kinetics was studied by measuring changes in current, charge, and mass on the working electrodes. Furthermore, the elementary reactions for each reduction reaction were discussed based on Tafel slopes. Accordingly, applied potential window for successful deposition of Te(0) was determined.

## Materials and Methods

All solutions were prepared by dissolving various amounts of tellurium dioxide (TeO_2_, 99+%, Acros Organics) in sodium hydroxide solutions (NaOH, 10 N, Fisher Chemical). The pH of each solution was adjusted by NaOH. All the solutions were deaerated by bubbling high-purity N_2_ (99.999 %) for 40 min. Linear sweep voltammetry was performed in a conventional three-electrode cell using a rotating disk electrode (RDE) [50-μm-thick Te films pre-deposited on gold-coated copper rods (diameter = 6.4 mm) embedded in a cylindrical Teflon holder] as working electrode, platinum-coated titanium stripe as counter electrode, and saturated Ag/AgCl as reference electrode. The scan rate was fixed at 1 mV/s.

Tafel plots were derived from LSV data. The effect of ${\text{TeO}}_{3}^{2-}$ concentration on the Tafel slope for the reduction reaction of Te(IV) was investigated by varying the ${\text{TeO}}_{3}^{2-}$(IV) concentration from 50 to 550 mM with a fixed solution pH of 12.0 at 23°C. Additionally, the effect of pH on the ${\text{TeO}}_{3}^{2-}$(IV) reduction reaction was investigated by varying the pH from 10.2 to 14.7 (calculated value) at a fixed ${\text{TeO}}_{3}^{2-}$ concentration of 550 mM at 23°C. The pH effect on the reduction reaction of Te(0) was investigated by varying the solution pH from 10.2 to 14.7, while fixing the ${\text{TeO}}_{3}^{2-}$ concentration and temperature at 0 mM and 23°C, respectively.

A potentiostat/galvanostat (Biologic, SP-200) combined with an EQCM (QCM200, Stanford Research Systems) was used for electrochemical investigation. The AT-cut 5-MHz quartz crystal covered with chromium/gold served as the working electrode. The platinum-coated titanium stripe and saturated Ag/AgCl were used as counter and reference electrodes, respectively. The effect of pH on reduction reaction of Te(IV) was examined in a range from 10.2 to 14.7 with ${\text{TeO}}_{3}^{2-}$ concentration of 300 mM at 23°C. To investigate the reduction reaction of Te(0) using EQCM at different pH (i.e., 10.2, 12.5, and 14.7), a thin film of Te with a mass of 70 ± 3.5 μg cm^−2^ was pre-deposited on the top of the working electrode. Scan rate of the EQCM experiments was 50 mV/s. Based on Sauerbrey equation, the frequency changes, Δ*f*, of the quartz crystal were correlated with the mass changes, Δ*m* (Seo et al., 1994):

(1) $$\begin{array}{l} \Delta f=-K\cdot \Delta m \end{array}$$

where *K* is the sensitivity factor for the crystal (i.e., 56.6 Hz μg^−1^ cm^2^).

The Faraday law can be described by the following equation:

(2) $$\begin{array}{l} Q=zn\Delta F \end{array}$$

where *Q* is the charge consumed during the deposition, *z* is the number of electrons involved in the reaction, Δ*n* is the change in the number of moles of the deposits, and *F* is the Faraday constant. When Equations (1) and (2) are combined with the equation Δ*m* = *M*·Δ*n*, in which *M* is molecular weight, the following equation can be derived (Saloniemi et al., 2000a,b):

(3) $$\begin{array}{l} \Delta m=\left(\frac{M}{zF}\right)Q \end{array}$$

In the correlation of Δ*m* as a function of *Q*, the slope (*S*) would then be defined as:

(4) $$\begin{array}{l} S=\frac{M}{zF} \end{array}$$

The number of the electrons involved in the reaction can be calculated by the following equation (Santos and Bulhoes, 2003):

(5) $$\begin{array}{l} \frac{M}{z}=S\cdot F \end{array}$$

## Results and Discussion

**Figure 1:**
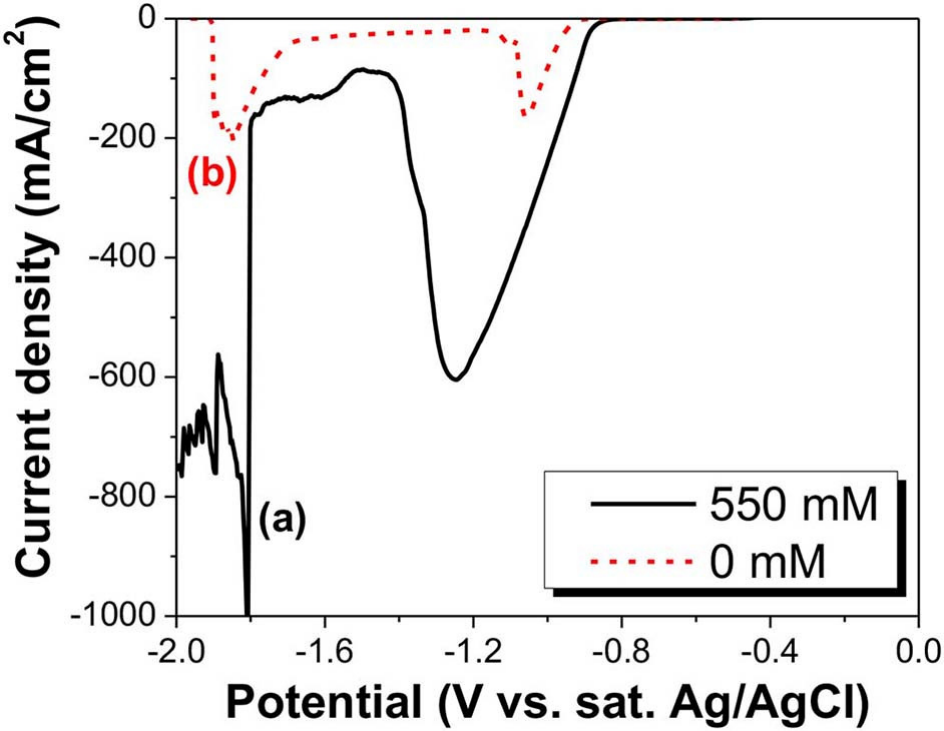
LSV curve of ${\text{TeO}}_{3}^{2-}$ in alkaline solutions at a pH of 14.7. The black solid curve is at a ${\text{TeO}}_{3}^{2-}$ concentration of 550 mM (a), and the red dashed curve is at a ${\text{TeO}}_{3}^{2-}$ concentration of 0 mM (b). Thick Te films were used as substrate.

The tellurium known to dissolve with simultaneous formation of ${\text{TeO}}_{3}^{2-}$(IV) and ${\text{Te}}_{2}^{2-}$(-I) ions in alkaline solution is cathodically reduced to give either Te(0) or Te^2−^(-II) ion. Therefore, a systematic voltammetric investigation using its polarographic behavior is required for a comprehensive understanding of Te reduction in alkaline media. As shown by the slight increase in the current density, the LSV curve (a) in Figure 1, the first reduction reaction started at −0.77 V. The current density continued to increase until the applied potential reached −1.25 V, where it marked a dipping point. The large increase in current density was probably caused by the reduction of ${\text{TeO}}_{3}^{2-}$, which are further discussed later in this paper. The current density at more negative potentials past the dip point started to rapidly decrease, and then, at −1.5 V, slightly increased again until the potential reached −1.8 V. The sharp spike in current density at −1.8 V might be attributed to hydrogen gas evolution reaction (Pourbaix, 1966). The curve (b) in Figure 1 was obtained by cathodic reduction of the Te(0) electrode in a NaOH solution in the absence of ${\text{TeO}}_{3}^{2-}$, in which the reduction reaction of Te(0)/${\text{Te}}_{2}^{2-}$(-I) started at −0.92 V. At more negative potentials, the Te(0)/${\text{Te}}_{2}^{2-}$(-I) reduction reaction rate increased, resulting in a sharp increase in the cathodic current density. However, at an applied potential of −1.06 V, the Te substrate was almost completely dissolved, leading to the rapid decrease in current density just beyond this point. The current density increased again at −1.7 V, but this was primarily due to H_2_ gas evolution (Reaction 7) (Pourbaix, 1966). E^0^ values of reactions 6–12 were calculated at pH of 12.5.

(6) $$\begin{array}{l} TeO_{3}^{2-}\left(aq\right)+3H_{2}O\left(l\right)+4e^{-}\to Te\left(s\right)+6OH^{-}\left(aq\right)\;\left({\text{E}}^{0}=-0.45\text{ V vs}.\text{Ag}/\text{AgCl}\right) \end{array}$$

(7) $$\begin{array}{l} 2H_{2}O\left(l\right)+2e^{-}\to H_{2}\left(g\right)+2OH^{-}\left(aq\right)\;\left({\text{E}}^{0}=-0.90\text{ V vs}.\text{Ag}/\text{AgCl}\right) \end{array}$$

(8) $$\begin{array}{l} 2Te\left(s\right)+2e^{-}\to Te_{2}^{2-}\left(aq\right)\;\left({\text{E}}^{0}=-0.91\text{ V vs}.\text{Ag}/\text{AgCl}\right) \end{array}$$

(9) $$\begin{array}{l} Te\left(s\right)+2e^{-}\to Te^{2-}\left(aq\right)\;\left({\text{E}}^{0}=-1.34\text{ V vs}.\text{Ag}/\text{AgCl}\right) \end{array}$$

(10) $$\begin{array}{l} Te_{2}^{2-}\left(aq\right)+2e^{-}\to 2Te^{2-}\left(aq\right)\;\left({\text{E}}^{0}=-1.47\text{ V vs}.\text{Ag}/\text{AgCl}\right) \end{array}$$

(11) $$\begin{array}{l} TeO_{3}^{2-}\left(aq\right)+2Te_{2}^{2-}\left(aq\right)+3H_{2}O\left(l\right)\to 5Te\left(s\right)+6OH^{-}\left(aq\right)\;\left({\text{G}}^{\text{O}}=-164.1\text{kJ}/\text{mol}\right) \end{array}$$

(12) $$\begin{array}{l} TeO_{3}^{2-}\left(aq\right)+2Te^{2-}\left(aq\right)+3H_{2}O\left(l\right)\to 3Te\left(s\right)+6OH^{-}\left(aq\right)\;\left(\Delta {\text{G}}^{\text{O}}=-279.3\text{kJ}/\text{mol}\right) \end{array}$$

**Figure 2:**
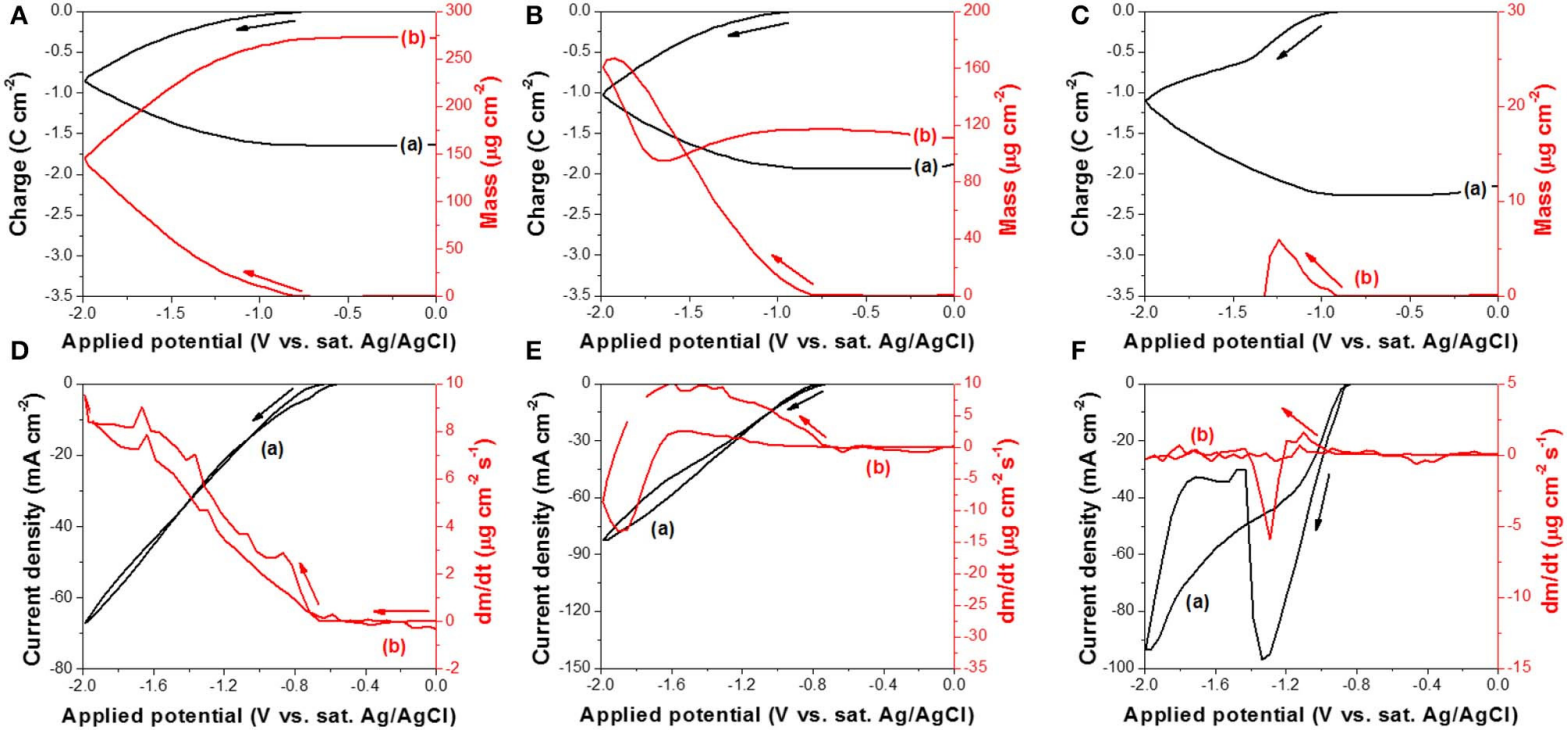
**(A–C)** are charge curve (a) calculated from cyclic voltammogram and cyclic gravimetric curve (b) measured by EQCM at different pH: **(A)** 10.2, **(B)** 12.5, and **(C)** 14.7. **(D–F)** are cyclic voltammogram (a) and mass change rate curve (b) measured by EQCM at different pH: **(D)** 10.2, **(E)** 12.5, and **(F)** 14.7. The experiments were conducted with 300 mM ${\text{TeO}}_{3}^{2-}$ and a scan rate of 50 mV/s at 23°C. The working electrode was commercialized Au/Cr crystal.

The reduction reaction of ${\text{TeO}}_{3}^{2-}$(IV) was further investigated by EQCM (Figure 2). Figures 2A–C show the change in charge and mass as a function of applied potential at different pH where the curve (a) represents the charge change and curve (b) represents the mass change. At a pH of 10.2 (Figure 2A), the mass increased monotonically as electrical charge increased, which meant Te was deposited onto the working electrode continuously. The mass change rate (Figure 2D, curve b) started to increase at −0.66 V and kept increasing monotonically as a function of applied potential until the voltage reached −0.86 V. The mass change rate started to fluctuate past the critical point because two reactions took place simultaneously in addition to Te deposition: hydrogen gas evolution and Te dissolution. In comparison to the Te dissolution observed at −0.97 V (Figure 1, curve b), the hydrogen gas evolution was probably the major reason for this fluctuation. Furthermore, the mass change rate remained positive after the critical point, meaning the rate of Te deposition was faster than that of Te dissolution. At a pH of 12.5 (Figure 2B), the mass of deposits increased with charge in the forward scan (i.e. −0.73 to −1.92 V). According to Figure 2E, the *dm/dt* started to increase at −0.73 V. At low overpotential, *dm/dt* (Figure 2E) increased monotonically when applied potential became more negative. At this range, Te(0) dissolution might have happened as well, but the rate of Te(0) dissolution was slower than the deposition rate. When the applied potential further decreased from −1.92 to −2.0 V or reversed from −2.0 to −1.66 V, the mass of Te deposits decreased. As shown in Figure 2E, the *dm/dt* started to decrease from less negative potentials than −1.92 V and then reached negative values as the dissolution rate exceeded that of the deposition. During the reverse scan, when applied potential is more positive than −1.66 V, the mass started to increase again as the Te deposition rate increased. At a pH of 14.7 (Figure 2C), the reduction reaction of ${\text{TeO}}_{3}^{2-}$(IV) started at an applied potential of −0.92 V. The mass of Te(0) continued to increase until −1.24 V, at which it quickly decreased all the way to zero by the time applied potential reached −1.31 V, caused by the fast dissolution of Te(0). As shown in Figure 2F curve (b), the mass change rate started decreasing at less negative potentials, but the total mass of Te(0) deposit did not reach its maximum until −1.24 V because the deposition rate by reduction of ${\text{TeO}}_{3}^{2-}$(IV) to Te(0) surpassed that of Te(0) dissolution. The curve (a) in Figure 2F also showed a current density dip at −1.34 V and then the current density decreased significantly. As shown by these observations, the formation of intermediate species [i.e., ${\text{Te}}_{2}^{2-}$(-I)] and its behavior as a function of pH differed the competition of dissolution and deposition rate. In alkaline solution, the reduction of Te(0) results in two possible species (i.e., ${\text{Te}}_{2}^{2-}$ and Te^2−^). These two products would react with ${\text{TeO}}_{3}^{2-}$(IV) (reactions 11 and 12), inhibiting its transport to the electrode surface and instead produce black Te particles in solution. The presence of such particles was observed right after re-exposing the gold substrate (Shinagawa et al., 1972; Wu et al., 2014). The curve (b) in Figure 2F showed that the mass change rate (*dm/dt*) increased slightly and then rapidly decreased, leading to a peak at −1.29 V, which meant that, in high pH, the rates of Te deposition and dissolution were affected dramatically as a function of overpotential. Thus, according to the EQCM data obtained at pH 14.7, the applied potential should be controlled from −0.92 to −1.31 V to deposit the Te on the surface of the working electrode. When the applied potential was from −1.31 to −2.00 V, the Te(0) was formed in the solution as a form of particles.

**Figure 3:**
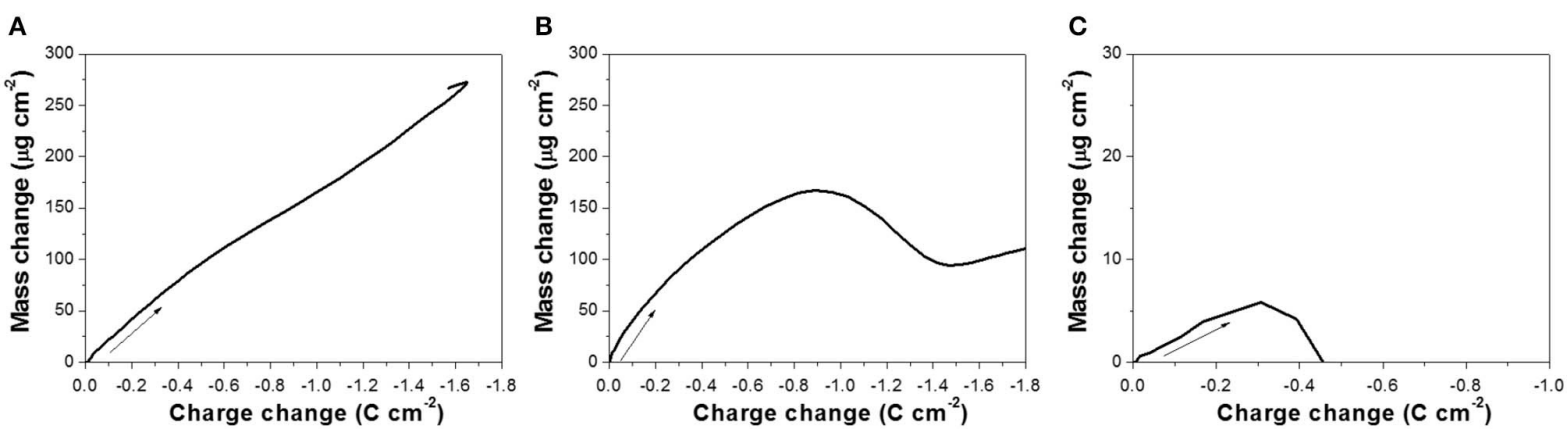
Mass change as a function of charge change, obtained from the mass variation profiles presented in Figure 2 at pH: **(A)** 10.2, **(B)** 12.5, and **(C)** 14.7, with 300 mM ${\text{TeO}}_{3}^{2-}$.

Based on the EQCM data, Δ*m* was plotted as a function of Δ*Q*, shown in Figure 3, and the slope (*S*) (Equation 4) for each curve was extracted to calculate the number of electrons involved in the reaction. To minimize the effect of secondary reaction, slopes were extracted based on the lower region of the curve. According to Equation (5), *M*/*z* was calculated to be 26.8, 35.5, and 1.9 g mol^−1^ at pH of 10.2, 12.5, and 14.7, respectively. Using the atomic number of Te(M_Te_) of 127.6 g mol^−1^, *z* at pH 10.2 and 12.5 was 4.7 and 3.6, respectively. From this calculation, it can be deduced that a four-electron reduction (Reaction 6) occurred and caused the increase of mass. Saloniemi et al. reported the *M*/*z* value of 22.8 g mol^−1^ in the solution of 1 mM ${\text{TeO}}_{3}^{2-}$ and 100 mM Na(CH_3_COO) at a pH of 9 on gold electrode, and they claimed that the reaction was a four-electron reduction of ${\text{TeO}}_{3}^{2-}$ to solid Te (Saloniemi et al., 2000b). However, at a pH of 14.7, the *z* value was much larger than 4, which might be attributed to the fact that the dissolution of Te (Reaction 8 or 9) started shortly after Reaction 6, and thus the relationship between Δ*m* and Δ*Q* (Figure 3C) was not only reflecting Reaction 6, but also Reaction 8 or 9 or even hydrogen gas evolution.

**Figure 4:**
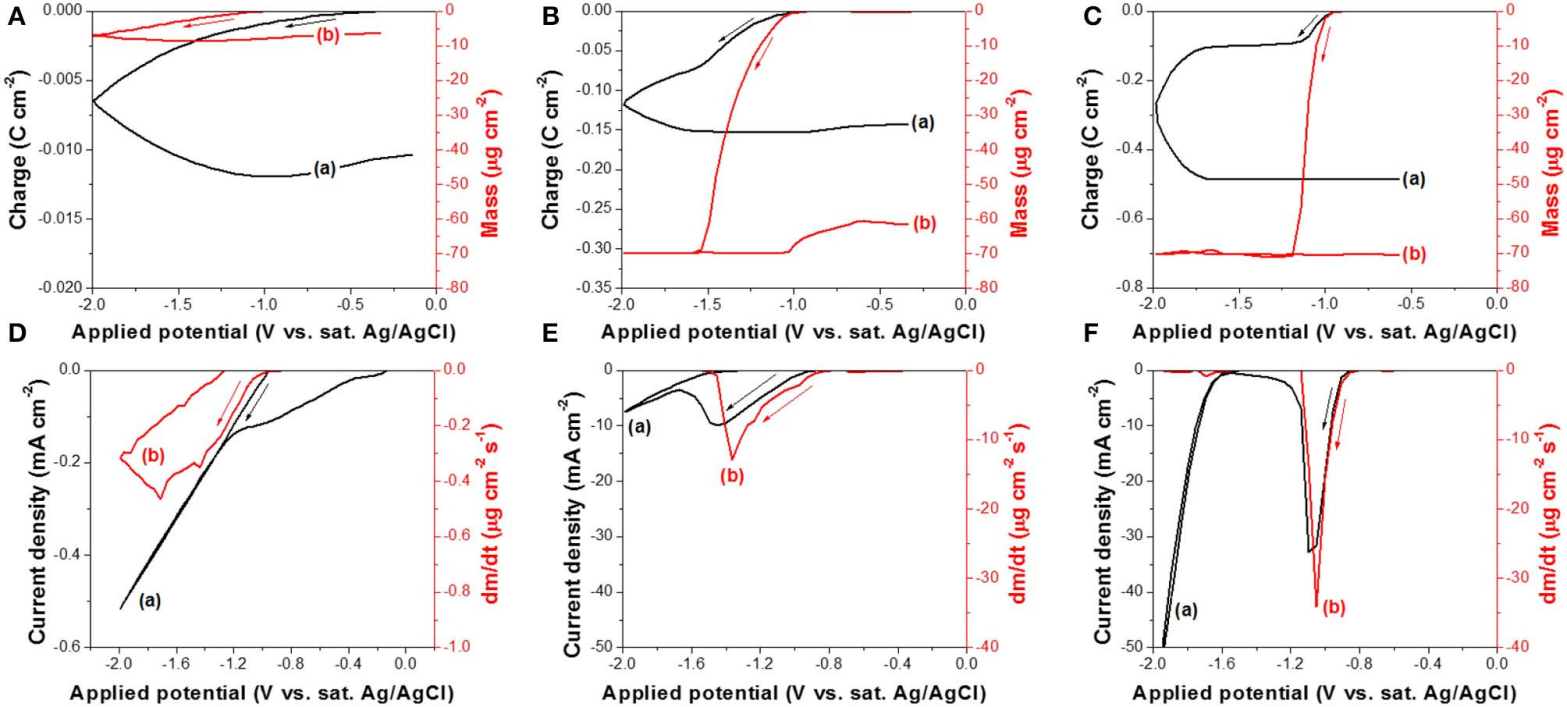
**(A–C)** are charge curve (a) calculated from cyclic voltammogram and cyclic gravimetric curve (b) measured by EQCM at different pH: **(A)** 10.2, **(B)** 12.5, and **(C)** 14.7. **(D–F)** are cyclic voltammogram (a) and mass charge rate curve (b) measured by EQCM at different pH: **(D)** 10.2, **(E)** 12.5, and **(F)** 14.7. The experiments were conducted with 0 mM ${\text{TeO}}_{3}^{2-}$ and a scan rate of 50 mV/s at 23°C. The working electrode is commercialized Au/Cr crystal coated with 70 ± 3.5 μg cm^−2^ Te thin film.

The reduction reaction of Te(0) to ${\text{Te}}_{2}^{2-}$(-I) or Te^2−^(-II) was also studied using EQCM at different pH (10.2, 12.5, and 14.7) (Figure 4). Before the EQCM experiments, a thin film of Te with a mass of 70 ± 3.5 μg/cm^2^ was electrodeposited onto the working electrode, and no ${\text{TeO}}_{3}^{2-}$ ions were present in the solution. The mass change was therefore negative, caused by the dissolution of Te(0) to ${\text{Te}}_{2}^{2-}$(-I) or Te^2−^(-II), which were solvable species. At a pH of 10.2 (Figure 4A), the mass of Te(0) started to decrease at −0.97 V during the forward scan, but the Te thin film was not completely dissolved even when the applied potential reached −2.0 V. The mass change rate (*dm/dt*) in Figure 4D was relatively small (−0.47 μg cm^−2^ s^−1^ at maximum) compared to the rate at a pH of 12.5 (−13.0 μg cm^−2^ s^−1^ at maximum) and 14.7 (−34.1 μg cm^−2^ s^−1^ at maximum). This suggested that OH^−^ or Na^+^ in the solution influenced the reaction rate significantly. At a pH of 12.5 (Figure 4B), the mass started to decrease at −1.0 V. When the applied potential was −1.54 V, the mass change was about −70 μg cm^−2^, which meant that all the pre-deposited Te thin film was dissolved. Figure 4E showed that the mass change rate (*dm/dt*) decreased to zero at −1.54 V, confirming there was no more Te(0) available to dissolve from the working electrode. The further increase in charge at more negative potentials than −1.54 V was probably caused by hydrogen gas evolution (Reaction 7) and possibly Reaction 10. At a pH of 14.7 (Figure 4C), the pre-deposited Te thin film started to dissolve at −0.95 V and was completely dissolved at −1.19 V. The mass change rate (*dm*/*dt*) at this pH was the highest of the three conditions, and it decreased to zero at −1.19 V (Figure 4F).

**Figure 5:**
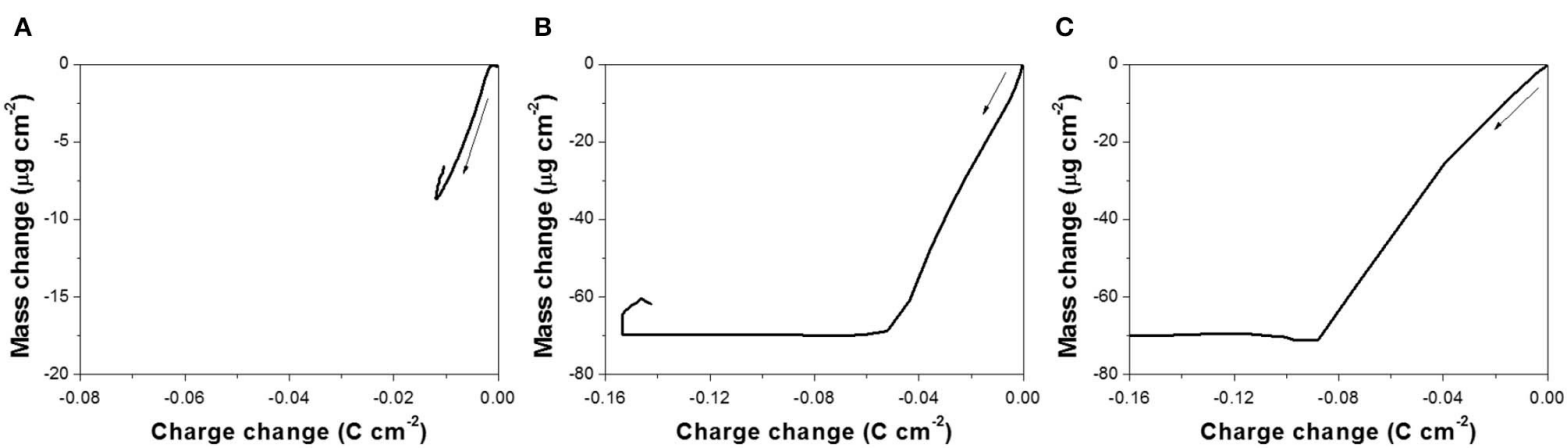
Mass change as a function of charge change, obtained from the mass variation profiles presented in Figure 4 at pH: **(A)** 10.2, **(B)** 12.5, and **(C)** 14.7, without ${\text{TeO}}_{3}^{2-}$.

Based on the EQCM data, the Δ*m* vs. Δ*Q* relationship was plotted in Figure 5. The *M*/*z* was calculated using the slope (*S*) extracted from each curve. The *M*/*z* values were 105.5, 126.3, and 76.9 g mol^−1^ at pH of 10.2, 12.5, and 14.7, respectively. The *z* values were then calculated using M_Te_ to be 1.21 and 1.01 at pH 10.2 and 12.5, respectively, which confirmed that the dissolution of Te(0) at pH 10.2 and 12.5 was a one-electron reduction of Te(0) to ${\text{Te}}_{2}^{2-}$ (Reaction 8). At a pH of 14.7, the *z* value was 1.66, so both Reaction 8—one-electron reduction—and Reaction 9—two-electron reduction—were possible. Based on the analysis in our previous publication, Te(0) was first reduced to ${\text{Te}}_{2}^{2-}$(-I) and then further reduced to Te^2−^(-II) in alkaline solution (Wu et al., 2015). Lingane and Niedrach also reported that Te(0) was reduced ${\text{Te}}_{2}^{2-}$(-I) first, then to Te^2−^(-II) (Lingane and Niedrach, 1949). At a pH of 14.7, these two reactions might have occurred sequentially. Although it was difficult to monitor the color change (${\text{Te}}_{2}^{2-}$ is purple, and Te^2−^ is colorless) due to the EQCM cell setup, the solution was nearly colorless after the reaction finished. Additionally, at a lower pH of 10.2 and 12.5, the ${\text{Te}}_{2}^{2-}$ ion would be relatively unstable so that it would split to Te(0) and Te^2−^(-I) by a disproportionation reaction. Therefore, it would be hard to be recorded as charge. Instead, this was confirmed by black deposits in the solution. As a result, the reduction of ${\text{TeO}}_{3}^{2-}$(IV) in alkaline solution would go through three steps: (1) Te(IV) reduced to Te(0), (2) Te(0) reduced to ${\text{Te}}_{2}^{2-}$(-I), and (3) ${\text{Te}}_{2}^{2-}$(-I) further reduced to Te^2−^(-II) (Lingane and Niedrach, 1949; Wu et al., 2015).

The onset potential was extracted from the LSV curves shown in Figures S1–S3. Figure S4 shows that the onset potential of ${\text{TeO}}_{3}^{2-}$(IV) to Te(0) became more positive when the concentration of Te(IV) ion (${\text{TeO}}_{3}^{2-}$) increased, but became more negative as a function of pH (Figure S5). Additionally, the onset potential of Te(0) to ${\text{Te}}_{2}^{2-}$ became more positive as a function of pH (Figure S6). For example, the onset potential of ${\text{TeO}}_{3}^{2-}$(IV) to Te(0) at pH of 12.5 and 14.7 are about −0.71 V and −0.85 V, respectively. The onset potential of Te(0) to ${\text{Te}}_{2}^{2-}$ at pH of 12.5 and 14.7 are about −1.02 V and −0.93 V, respectively. Therefore, the window to possibly deposit compact Te film at pH of 12.5 and 14.7 is 0.31 V and 0.08 V, respectively. Because ${\text{Te}}_{2}^{2-}$ is a solvable species in alkaline solution, once the reaction of Te(0) to ${\text{Te}}_{2}^{2-}$ happened, the morphology of the Te film would be deteriorated significantly. From this observation, the morphology of Te electrodeposits in alkaline bath would be highly dependent on the tellurite ion concentration and pH.

**Figure 6:**
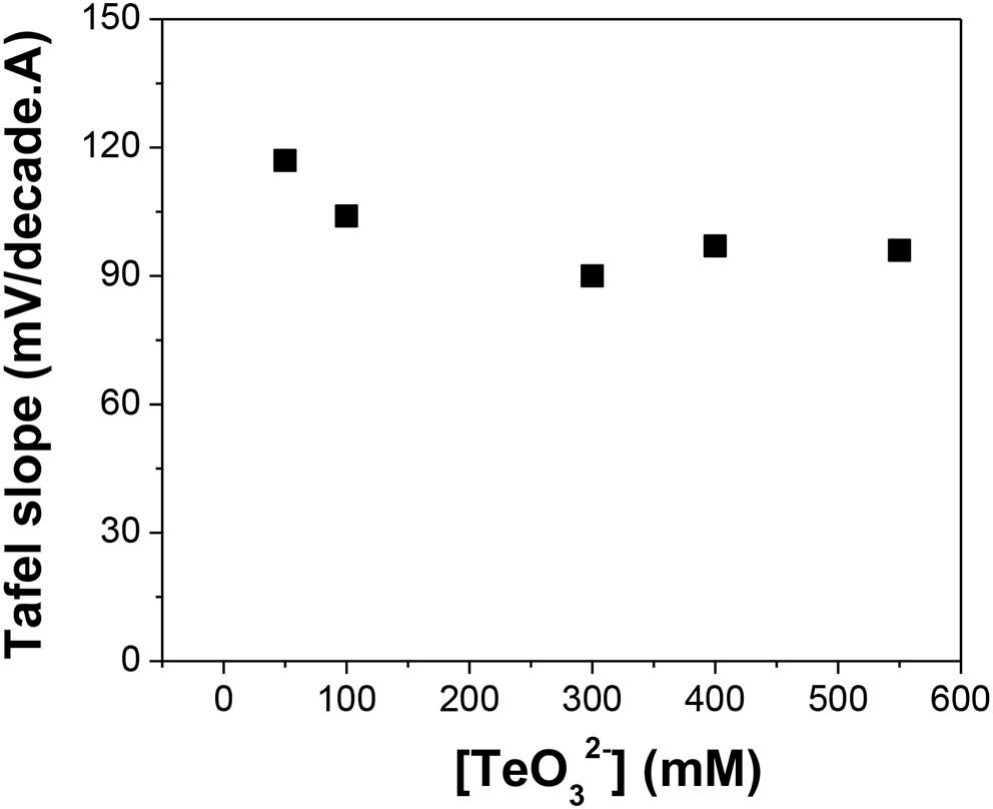
Tafel slope of ${\text{TeO}}_{3}^{2-}$ reduction reaction in alkaline solutions with different [${\text{TeO}}_{3}^{2-}$]: 50, 100, 300, 400, and 550 mM. The experiments were conducted using Te as a substrate at pH of 12.0 and temperature of 23°C.

**Figure 7:**
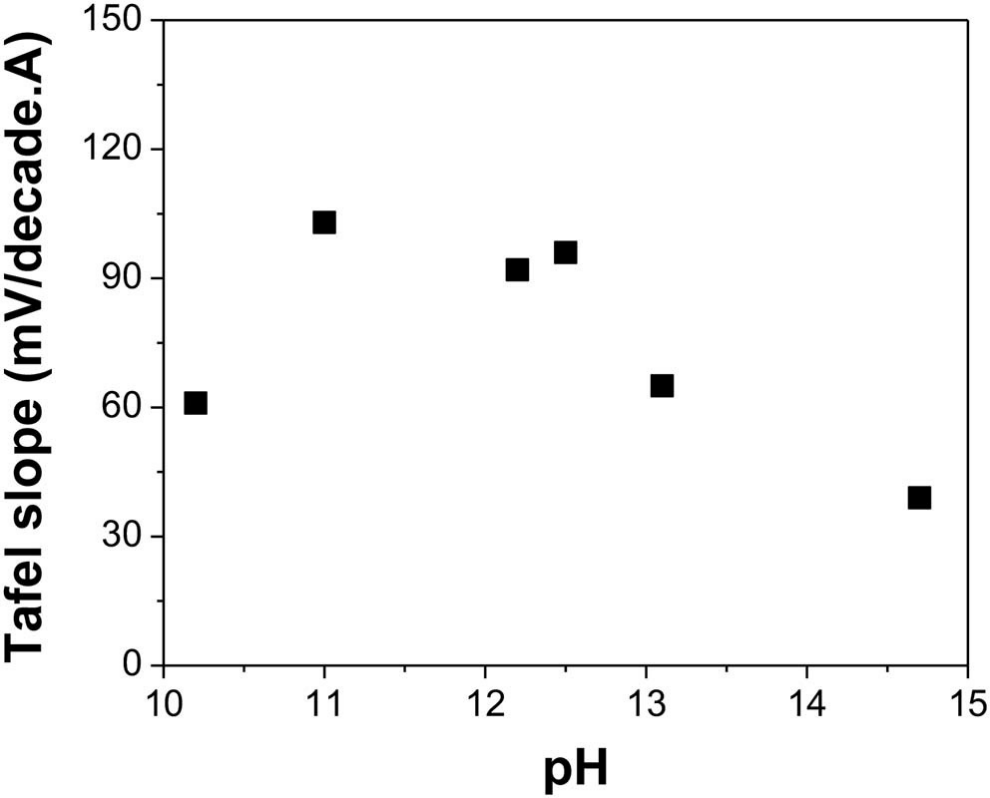
Tafel slope of ${\text{TeO}}_{3}^{2-}$ reduction reaction in alkaline solutions with different pH: 10.2, 11.0, 12.2, 12.5, 13.1, and 14.7 (calculated value). The experiments were conducted using Te as a substrate at [${\text{TeO}}_{3}^{2-}$] of 550 mM and a temperature of 23°C.

**Figure 8:**
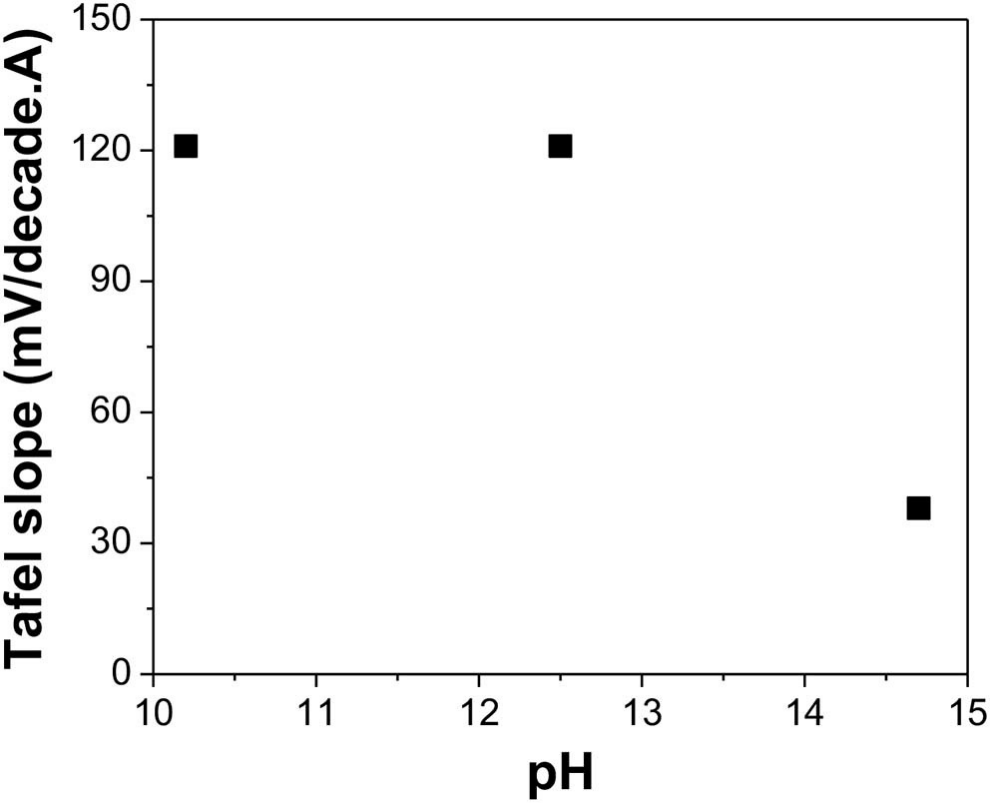
Tafel slope of Te(0) reduction reaction in alkaline solutions with different pH: 10.2, 12.5, and 14.7 (calculated value). The experiments were conducted using Te as a substrate at [${\text{TeO}}_{3}^{2-}$] of 0 mM and a temperature of 23°C.

The Tafel slopes of reduction reactions of Te(IV) to Te(0) (Figures 6, 7) and of Te(0) to ${\text{Te}}_{2}^{2-}$(-I) (Figure 8) were extracted from the Tafel plots (Figures S7–S9). In alkaline solution, it is known that the Te(IV) exists in the form of anion [e.g., ${\text{TeO}}_{3}^{2-}$; Bard, 1975, ${\text{Te}(\text{OH})}_{6}^{2-}$; Komandenko and Rotinyan (1967b)]. According to our previous work, the direct reduction of ${\text{TeO}}_{3}^{2-}$(IV) to Te(0) unlikely proceeded on a negatively charged cathodic electrode surface (Wu et al., 2015). Therefore, a four-step process is proposed through the sequence of Reactions 14–17. Reaction 13 shows that the anions ${\text{Te}(\text{OH})}_{6}^{2-}$ are in equilibrium with ${\text{Te}(\text{OH})}_{3}^{+}$ cations. Once the ${\text{Te}(\text{OH})}_{3}^{+}$ is adsorbed on the cathodic electrode surface, it obtains an electron from the electrode and is then discharged as Te(OH)_3_. This cyclic mechanism of picking up an electron from the electrode is repeated in Reactions 15 and 16, yielding ${\text{Te}(\text{OH})}_{3}^{2-}$. The ${\text{Te}(\text{OH})}_{3}^{2-}$ obtains a fourth and last electron in Reaction 17 and releases all three hydroxide ions to form Te element. According to the Tafel equation, by assuming a charge transfer coefficient of 0.5, the Tafel slopes are 118, 59, 39, and 30 mV/decade when Reactions 14, 15, 16, and 17 are the rate-limiting steps, respectively (Bard and Faulkner, 2001). Komandenko and Rotinyan reported that under their experimental conditions, Reaction 16 was the rate-limiting step, and the Tafel slopes ranged from 40 to 45 mV/decade (Komandenko and Rotinyan, 1966). Furthermore, they claimed that at low alkali concentration, the ${\text{Te}(\text{OH})}_{3}^{+}$ would directly obtain two electrons and become ${\text{Te}(\text{OH})}_{3}^{-}$. Thus, they replaced Reactions 14 and 15 with one reaction (Komandenko and Rotinyan, 1967a).

Figure 6 shows that when the Te precursor concentration increased from 50 to 550 mM at a pH of 12.0, the Tafel slopes ranged from 90 to 117 mV/decade. The differences between Tafel slopes were attributed to changes in the charge transfer coefficient. This meant that the ${\text{TeO}}_{3}^{2-}$ concentration did not influence the rate-limiting step substantially. In the whole concentration range, the rate-limiting step was Reaction 14, which was the discharge of ${\text{Te}(\text{OH})}_{3}^{+}$.

Figure 7 indicates that at low pH (i.e., 10.5), the Tafel slope was 61 mV/decade, which meant that Reaction 15 was the rate-limiting step. However, when the pH was varied between 11.0 and 12.5, the Tafel slopes of the reaction ranged from 92 to 103 mV/decade, which correlated to Reaction 14 as the rate-limiting step. When pH increased to 13.1, the Tafel slope shifted to 65 mV/decade, making Reaction 15 the rate-limiting step. When pH further increased to 14.7, the Tafel slope was further reduced to 39 mV/decade, indicating that Reaction 16 was the rate-limiting step.

(13) $$\begin{array}{l} Te{\left(OH\right)}_{6}^{2-}\left(aq\right)\to Te{\left(OH\right)}_{3}^{+}\left(aq\right)+{3OH}^{-}\left(aq\right) \end{array}$$

(14) $$\begin{array}{l} Te{\left(OH\right)}_{3}^{+}\left(aq\right)+e^{-}\to Te{\left(OH\right)}_{3}\left(ads\right) \end{array}$$

(15) $$\begin{array}{l} Te{\left(OH\right)}_{3}\left(ads\right)+e^{-}\to Te{\left(OH\right)}_{3}^{-}\left(ads\right) \end{array}$$

(16) $$\begin{array}{l} Te{\left(OH\right)}_{3}^{-}\left(ads\right)+e^{-}\to Te{\left(OH\right)}_{3}^{2-}\left(ads\right) \end{array}$$

(17) $$\begin{array}{l} Te{\left(OH\right)}_{3}^{2-}\left(ads\right)+e^{-}\to Te\left(s\right)+{3OH}^{-}\left(aq\right) \end{array}$$

The Tafel slopes for reduction of Te(0) to ${\text{Te}}_{2}^{2-}$(-I) are shown in Figure 8. At pH of 10.2, 12.5, and 14.7, the Tafel slopes were about 121, 121, and 38 mV/decade, respectively. The single Tafel slope of Te(0) to ${\text{Te}}_{2}^{2-}$(-I) was first reported to be 100 mV/decade at low NaOH concentration by Awad (1961). At high NaOH concentrations, two linear regions were observed with Tafel slopes of about 40 and 100 mV/decade. They proposed a two-step reaction mechanism, which involved Na^+^ ions (Reactions 18 and 19). However, the calculated number of electrons transferred for each elementary reaction was surprisingly two electrons for Reaction 18 and four electrons for Reaction 19, which could not be explained by this two-step reaction mechanism.

(18) $$\begin{array}{l} Te\left(s\right)+Na^{+}\left(aq\right)+e^{-}\to NaTe\left(ads\right) \end{array}$$

(19) $$\begin{array}{l} NaTe\left(ads\right)+Te\left(s\right)+e^{-}\to Te_{2}^{2-}\left(aq\right)+Na^{+} \end{array}$$

Therefore, Awad proposed a four-step reaction mechanism (Reactions 20–23) and introduced diatomic tellurium. In the reactions, M^+^ represented the cations (e.g., Na^+^ or Ba^2+^) in the alkaline solution. Reactions 20 and 21 were discharge reactions; Reactions 22 and 23 were electrochemical reactions. In the two discharge reactions, the reaction rate of the second step (Reaction 21) was relatively slower than that of the first step, which could be the rate-limiting step. On the other hand, in the two electrochemical reactions, the reaction rate of the second step (Reaction 23) was relatively slower than that of the first step, which could be the rate-limiting step. Awad used the energy barrier diagram of these four reactions to prove their assumptions. According to the energy barrier diagram, the first discharge (Reaction 20) and electrochemical (Reaction 22) steps had very low energy barriers compared to the second discharge (Reaction 21) and electrochemical (Reaction 23) steps. When Reaction 21 was the rate-limiting step, the Tafel slope was about 100 mV/decade, which was consistent with the theoretically calculated value of 120 mV/decade, taking into account the mechanism and the differences due to small changes in the charge transfer coefficient. When Reaction 23 was the rate-limiting step, the Tafel slope was about 40 mV/decade at low current density and 100 mV/decade at high current density. Therefore, in weakly alkaline solutions, the second discharge reaction (Reaction 21) was the rate-limiting step throughout the whole current density range with a Tafel slope of 100 mV/decade, while in strong alkaline solutions, the second electrochemical reaction (Reaction 23) was the rate-limiting step with a Tafel slope of 40 mV/decade at low current density and a Tafel slope of 100 mV/decade at high current density (Awad, 1962).

(20) $$\begin{array}{l} Te_{2}\left(s\right)+M^{+}\left(aq\right)+e^{-}\to MTe_{2}\left(ads\right) \end{array}$$

(21) $$\begin{array}{l} MTe_{2}\left(ads\right)+M^{+}\left(aq\right)+e^{-}\to {M_{2}Te}_{2}\left(ads\right) \end{array}$$

(22) $$\begin{array}{l} Te_{2}\left(s\right)+{M_{2}Te}_{2}\left(ads\right)+e^{-}\to M_{2}Te_{4}^{-}\left(aq\right) \end{array}$$

(23) $$\begin{array}{l} M_{2}Te_{4}^{-}\left(aq\right)+e^{-}\to 2Te_{2}^{2-}\left(aq\right)+2M^{+}\left(aq\right) \end{array}$$

Another mechanism was suggested by Komandenko and Rotinyan with Tafel slopes of about 40 mV/decade for the reduction of Te(0) to ${\text{Te}}_{2}^{2-}$(-I) at moderately alkaline solutions. Also, they proposed a different mechanism, which involved OH^−^ ions shown in Reactions 24–27 where Reaction 25 was the rate-limiting step (Komandenko and Rotinyan, 1967b).

(24) $$\begin{array}{l} Te\left(s\right)+OH^{-}\left(aq\right)+e^{-}\to TeOH^{2-}\left(ads\right) \end{array}$$

(25) $$\begin{array}{l} TeOH^{2-}\left(ads\right)+Te\left(s\right)+e^{-}\to {Te_{2}OH}^{3-}\left(ads\right) \end{array}$$

(26) $$\begin{array}{l} {Te_{2}OH}^{3-}\left(ads\right)+Te\left(s\right)+e^{-}\to {Te_{3}OH}^{4-}\left(ads\right) \end{array}$$

(27) $$\begin{array}{l} {Te_{3}OH}^{4-}\left(ads\right)+Te\left(s\right)+e^{-}\to 2Te_{2}^{2-}\left(aq\right)+OH^{-}\left(aq\right) \end{array}$$

Figure 8 shows that at relatively weak alkaline solutions (i.e., pH ≤ 12.5), the Tafel slope was about 120 mV/decade. This could be explained by Awad's mechanism, in which the second discharge reaction (Reaction 21) was the rate-limiting step (Awad, 1962). At strong alkaline solutions (i.e., pH 14.7), the Tafel slope was 38 mV/decade at low current density, which meant that the second electrochemical reaction (Reaction 23) was the rate-limiting step. However, at high current density, the Tafel slope remained at 40 mV/decade instead of 100 mV/decade.

## Conclusion

The electrochemical reduction mechanism and kinetics of ${\text{TeO}}_{3}^{2-}$(IV) and Te(0) were investigated by LSV and EQCM. These data indicated that reduction of ${\text{TeO}}_{3}^{2-}$(IV) to Te(0) is a four-electron reaction, while the reduction of Te(0) to ${\text{Te}}_{2}^{2-}$(I) is a single-electron reaction. Furthermore, the applied potential must tune to deposit Te(0) on the electrode surface, and it varied as a function of pH. When the applied potential was too negative, Te(0) nanoparticles were formed in the solution instead of on the electrode surface. As the pH increased, the reduction rate of Te(0) to ${\text{Te}}_{2}^{2-}$(-I) was accelerated. Additionally, investigation of two Te reduction reactions [${\text{TeO}}_{3}^{2-}$(IV) to Te(0) and Te(0) to ${\text{Te}}_{2}^{2-}$(-I)] showed that although the concentration of ${\text{TeO}}_{3}^{2-}$ affected mass transfer and reaction rates, it did not influence the rate-limiting step. Instead, solution pH controlled the reaction mechanism. At relatively weak alkaline solutions (i.e., pH ≤ 12.5), the discharge of ${\text{Te}(\text{OH})}_{3}^{+}$ was the rate-limiting step. For the reduction of Te(0) to ${\text{Te}}_{2}^{2-}$(-I), the second discharge reaction was the rate-limiting step with the Tafel slope of 120 mV/decade. At strong alkaline solutions (i.e., pH 14.7), the Tafel slope was 40 mV/decade at low current density, which meant that the second electrochemical reaction was the rate-limiting step.

## Data Availability Statement

All datasets generated for this study are included in the article/Supplementary Material.

## Author Contributions

TW, JK, and NM evenly contributed to the conception or design of the work, or the acquisition, analysis, or interpretation of data for the work. Also, drafting the work or revising it critically for experimental results.

### Conflict of Interest

The authors declare that the research was conducted in the absence of any commercial or financial relationships that could be construed as a potential conflict of interest.
