# Supplementary material for: Electrochemical Mechanism of Tellurium Reduction in Alkaline Medium
Source: Front Chem. 2020 Mar 3;8:84. doi: 10.3389/fchem.2020.00084 (PMC7062915; doi:10.3389/fchem.2020.00084)
Supplement: Supplementary file 1 [file Data_Sheet_1.pdf]

Supplementary Material

1 Supplementary Figures

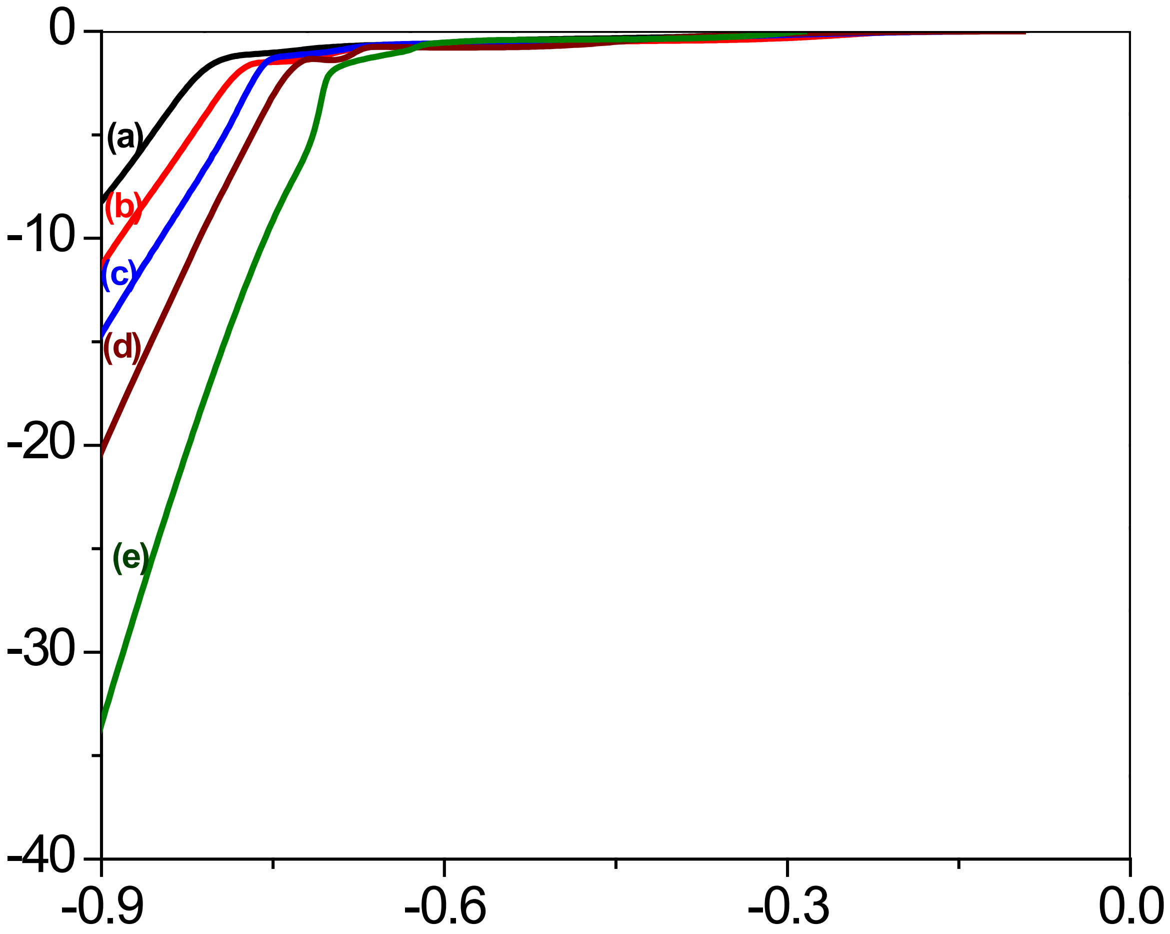

Figure S1. LSV curve of TeO_3^{2-} in alkaline solutions with different TeO_3^{2-} concentration: (a) 25, (b) 50, (c) 100, (d) 300 and (e) 550 mM at pH of 12.0 and temperature of 23 °C. Thick Te films were used as substrate.

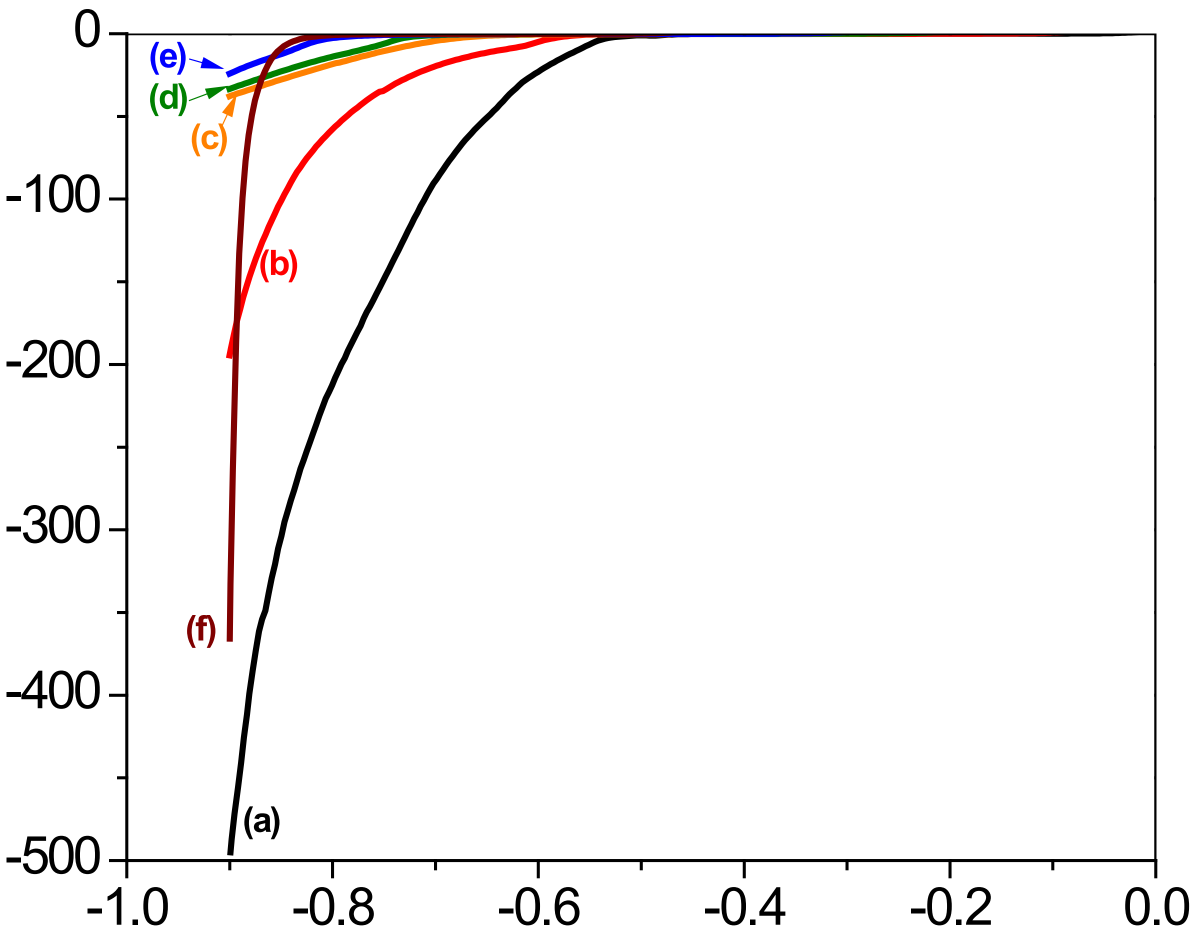

Figure S2. LSV curve of TeO_3^{2-} in alkaline solutions at different pH: (a) 10.2, (b) 11.0, (c) 12.2, (d) 12.5, (e) 13.1 and (f) 14.7. TeO_3^{2-} concentration was fixed at 550 mM and temperature was fixed at 23 °C. Thick Te films were used as substrate.

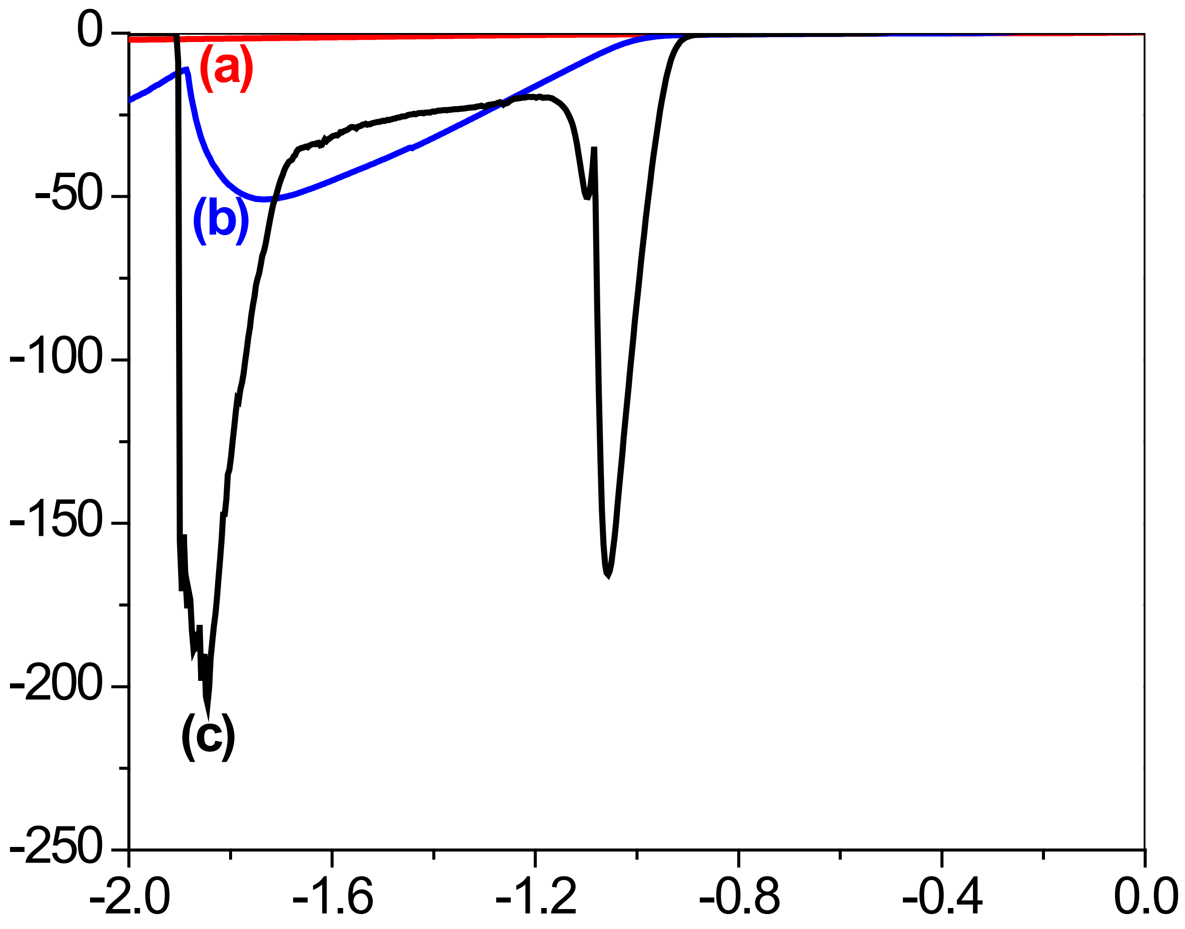

Figure S3. LSV curve of Te in alkaline solutions at different pH: (a) 10.2, (b) 12.5 and (c) 14.7. TeO_3^{2-} concentration and temperature were fixed at 0 mM and 23 °C, respectively. Thick Te films were used as substrate.

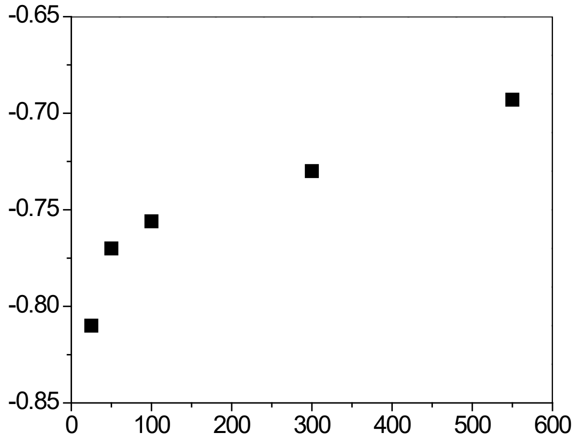

Figure S4. Onset potential of TeO_3^{2-} reduction reaction in alkaline solutions with different TeO_3^{2-} concentration (25, 50, 100, 300 and 550 mM) at pH of 12.0 and temperature of 23 ºC. Thick Te films were used as substrate.

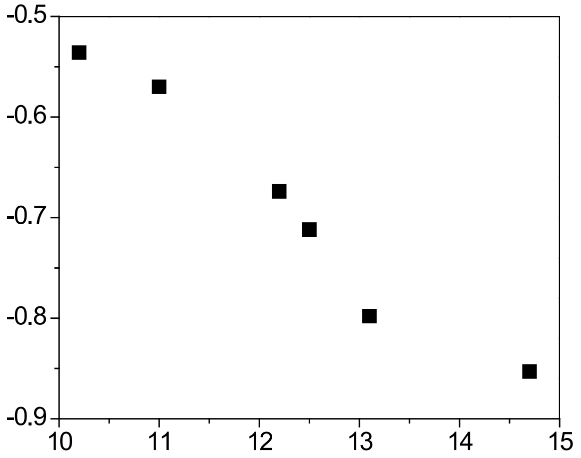

Figure S5. Onset potential of TeO_3^{2-} reduction reaction in alkaline solutions at different pH (10.2, 11.0, 12.2, 12.5, 13.1 and 14.7). TeO_3^{2-} concentration was fixed at 550 mM and temperature was fixed at 23 ºC. Thick Te films were used as substrate.

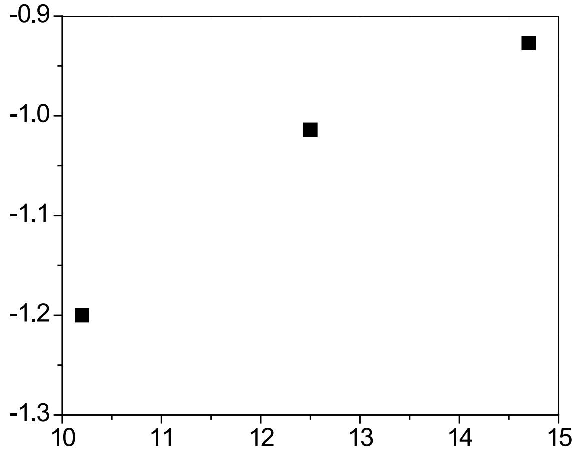

Figure S6. Onset potential of Te reduction reaction in alkaline solutions at different pH (10.2, 12.5 and 14.7). TeO_3^{2-} concentration was 0 mM and temperature was fixed at 23 °C. Thick Te films were used as substrate.

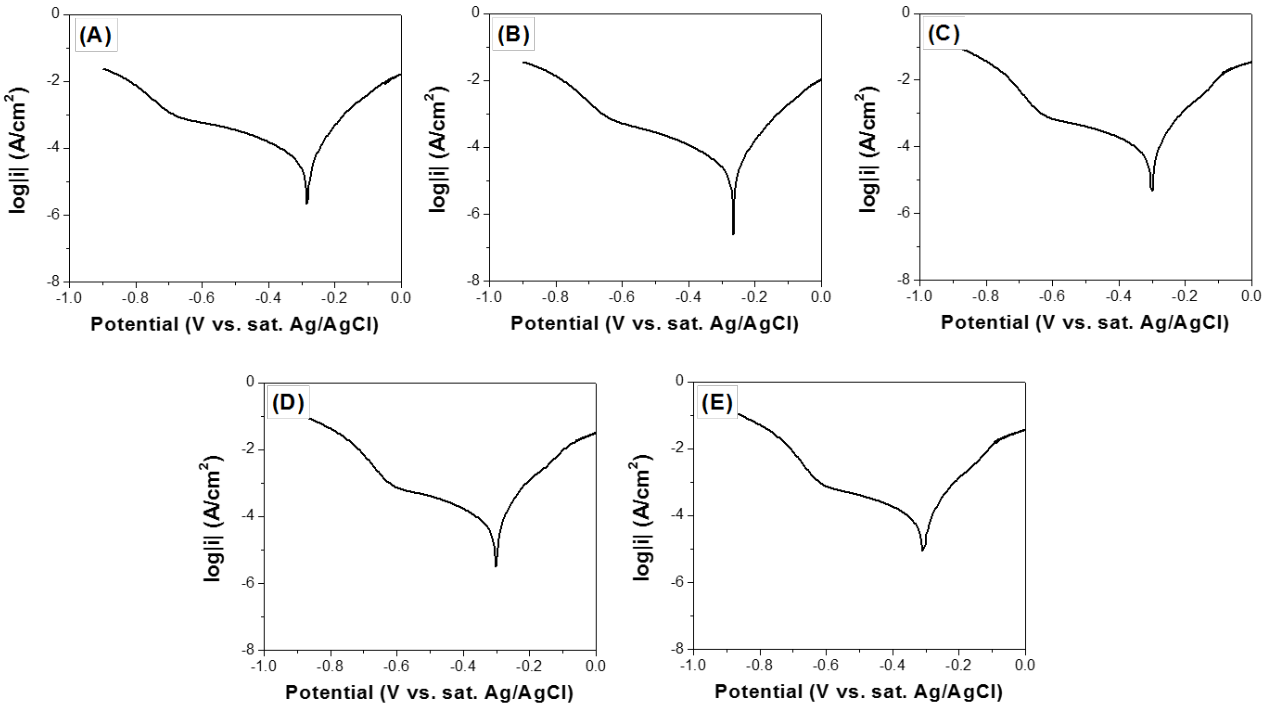

Figure S7. Tafel plot of TeO_3^{2-} reduction in alkaline solutions with different [TeO_3^{2-}]. (A) 50, (B) 100, (C) 300, (D) 400, (E) 550 mM. The experiments were conducted using Te as a substrate at pH of 12.0 and temperature of 23 ºC.

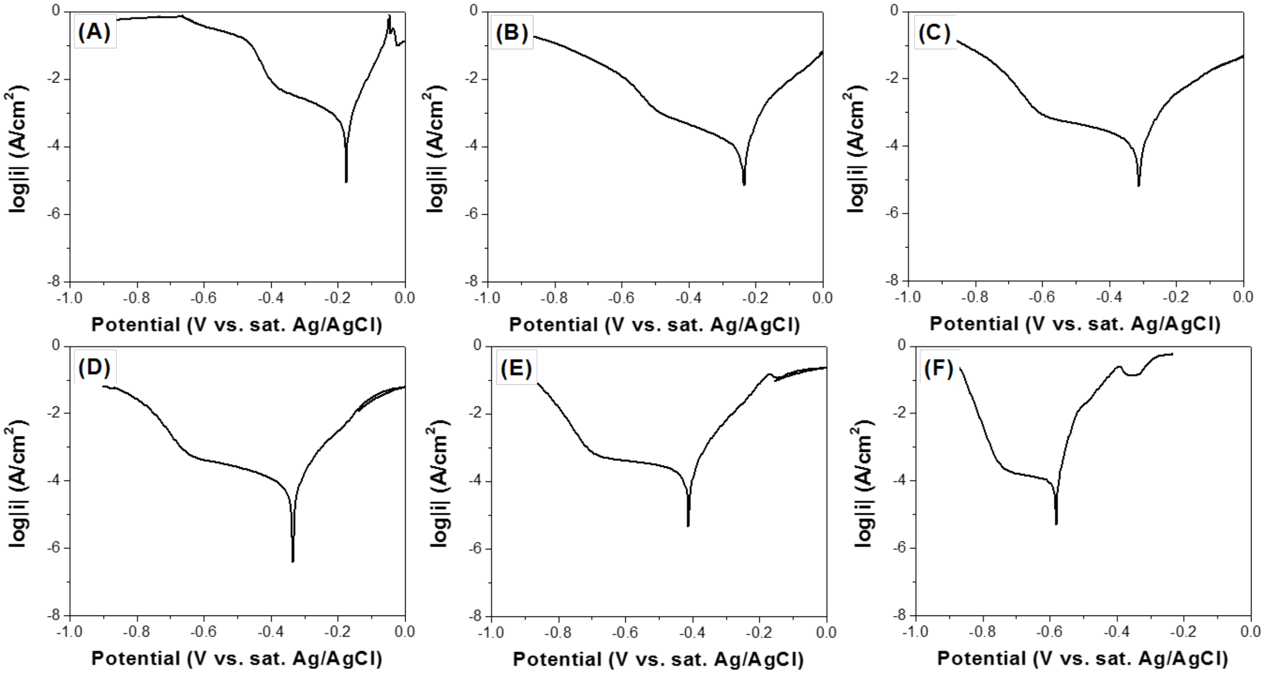

Figure S8. Tafel plot of TeO_3^{2-} reduction in alkaline solutions with different pH: (A) 10.2, (B) 11.0, (C) 12.2, (D) 12.5, (E) 13.1, (F) 14.7 (calculated value). The experiments were conducted using Te as a substrate at [TeO_3^{2-}] of 550 mM and temperature of 23 ºC.

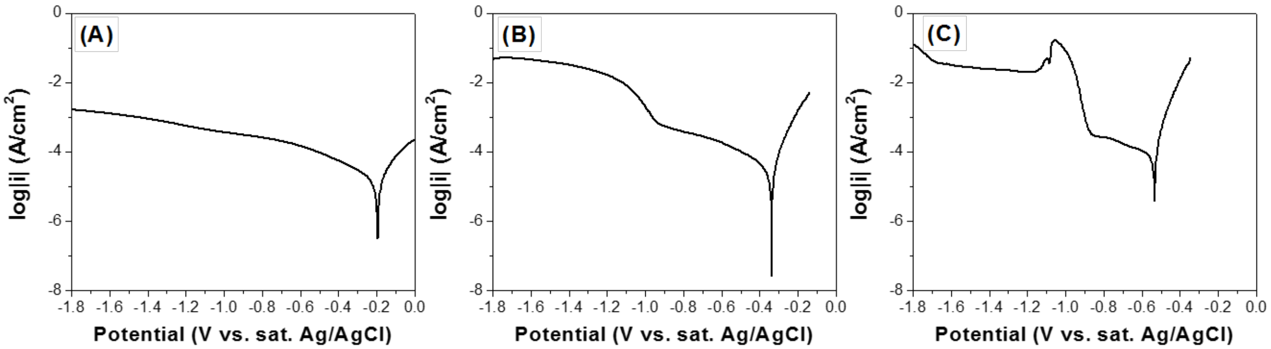

Figure S9. Tafel plot of Te dissolution in alkaline solutions with different pH: (A) 10.2, (B) 12.5 and (C) 14.7(calculated value). The experiments were conducted using Te as a substrate at [TeO_3^{2-}] of 0 mM and temperature of 23 ºC.
